# Editorial: Innovative solutions for host immunomodulation

**DOI:** 10.3389/fimmu.2026.2001079

**Published:** 2026-09-29

**Authors:** Namita Rout, Mallary Greenlee-Wacker, Shanjana Awasthi

**Affiliations:** 1 Tulane National Biomedical Research Center, Tulane School of Medicine- Department of Microbiology and Immunology, Covington, LA, United States; 2 Department of Biological Sciences, California Polytechnic State University, San Luis Obispo, CA, United States; 3 Department of Pharmaceutical Sciences, University of Oklahoma Health Campus, Oklahoma City, OK, United States

**Keywords:** host-pathogen interactions, immunomodulation, innate immunity, multidisciplinary approaches, therapeutic development

Recent advances across multidisciplinary fields and emerging technologies have enabled the identification of novel molecular targets and key signaling pathways that regulate host immunobiology in infectious, inflammatory, and cancer-related diseases. These developments have created new opportunities to precisely modulate immune responses, minimize collateral tissue damage, and develop innovative therapeutic strategies with translational and clinical potential. This Research Topic is organized to highlight the latest advances in understanding the mechanistic basis of host immunobiology and their implications for the development of targeted therapeutic interventions.

The collection comprises six articles, including five original research articles and one review article. Focusing on mechanisms of immune regulation and therapeutic innovation, these studies provide insights into treating infection, inflammation, and cancer. In the following section, we highlight the major findings of each article included in this Research Topic.

Yang et al. characterized pathogen-specific and shared host responses in sepsis with particular emphasis on complement/coagulation-related pathways, platelet-driven thromboinflammation, proteostasis-related stress, oxidative stress, and metabolic remodeling. Sepsis management requires shifting from generic anti-inflammatory approaches to pathogen-specific immunometabolic strategies. By profiling the plasma proteome and metabolome in *Escherichia coli* and *Klebsiella pneumoniae* bloodstream infections (BSI), this multi-omics study demonstrated that while both pathogens trigger shared thromboinflammatory cascades, *E. coli* drove a platelet-centered phenotype, whereas *K. pneumoniae* altered proteostasis and lipid metabolism. Persistent metabolomic divergence post-covariate adjustment highlighted small-molecule dynamics as sensitive host-stress markers. Leveraging these signatures, an exploratory four-protein panel (FCGR3A, COPS3, NT5C3A, ADO) offered preliminary discrimination for *E. coli* BSI. Nonetheless, given the baseline clinical diversity and modest cohort size, these initial insights warrant prospective validation in broader, independent populations.

Wang et al. investigated the immunomodulatory activity of *Codonopsis pilosula* (a natural plant)-derived polysaccharide (CPPS) using outer membrane protein 19 (OMP19) from *Brucella abortus* as the inflammatory stimulus. In a murine pretreatment model, CPPS attenuated OMP19-induced tissue damage and decreased the expression of HMGB1, E-cadherin and paxillin. *In vitro*, CPPS suppressed pro-inflammatory cytokine transcription, inhibited SYK/FAK/AKT phosphorylation, reduced PKC and WNT1 levels, downregulated E-cadherin and paxillin expression, and lowered intracellular Ca²^+^ concentrations. Transcriptomic and knockdown studies identified ATP2A1, an endoplasmic reticulum Ca²^+^-ATPase, as a key mediator of CPPS activity against OMP19. However, because the study did not include a live *Brucella* infection model or a therapeutic treatment paradigm, the relevance of these findings to brucellosis remains to be determined.

Xin et al. highlighted the role of TRIM28 in host-viral interactions and antiviral immune responses. The authors demonstrated that TRIM28 functions as a negative regulator of RIG-I-mediated responses by facilitating K48-linked polyubiquitination and proteasomal degradation of mitochondrial antiviral signaling protein (MAVS) at K136 and K461 residues *via* its N-terminal domain. The study advances prior work by proving the *in vivo* physiological relevance of this checkpoint and uncovering a viral infection-triggered, CRM1-dependent nuclear export of TRIM28. Cytoplasmic TRIM28 suppressed TBK1/IRF3 activation, whereas lung-specific TRIM28 knockdown restored type I interferon production and restricted viral replication *in vivo*. The TRIM28-MAVS axis thus highlights post-translational checkpoints as promising targets for host-directed antiviral therapies.

De Silva et al. investigated photodynamic priming to modulate the tumor microenvironment and responsiveness to checkpoint blockade. This approach uses a light-activated compound to induce immunogenic cell death and enhance anti-tumor immunity. Here, the authors exposed organoids from patients with pancreatic ductal adenocarcinoma (PDAC), a malignancy with an immunosuppressive and fibrotic phenotype, to Visudyne and red light. Consistent with the intended immunomodulatory effect, the photodynamic priming therapy increased cytotoxicity, upregulated damage-associated molecular pattern (DAMP) expression, and reduced immunosuppressive markers such as TGF-β and PD-L1. Additionally, dendritic cells (DCs) exposed to photo-primed organoids expressed more antigen-presenting molecules and effectively primed T cells than DCs exposed to untreated organoids. Anti-tumor T cell responses to photo-primed organoids were further amplified with checkpoint blockade, highlighting the potential for combined immunomodulatory therapy in PDAC.

Węgierek-Ciura et al. evaluated strategies to counter tumor-induced immunosuppressive microenvironment using cytokine-engineered DC vaccines, anti-IL-10 receptor (anti-IL-10R) antibodies, and hydroxyethyl starch–methotrexate (HES-MTX) nanoconjugate chemotherapy. Because IL-10 suppresses DC maturation and antitumor immunity, the authors evaluated multi-component regimens to restore effective responses. The tumor growth inhibition depended on the type of DC vaccine. The combination of two-component IL-12- and IL-18-DC vaccine plus anti-IL-10R antibodies was most effective without chemotherapy. When pretreatment with the HES-MTX chemotherapeutic agent was included, three-component IL-12-, IL-15- and IL-18-DC vaccine plus anti-IL-10R antibodies were most effective in tumor inhibition. Overall, the multi-component chemoimmunotherapy strategies enhanced antitumor effector cells with simultaneous reduction in immunosuppressive regulatory T cells and tumor-associated macrophages. These results obtained in a murine melanoma model provide a rationale for evaluating integrated chemoimmunotherapeutic strategies, combining DC vaccines, cytokine-pathway modulation, and immunomodulatory chemotherapy.

Firouzi et al. provide a comprehensive review on the role of immune cell-derived exosomes in regulating tumor-immune interactions. Instead of cataloging exosome or extracellular vesicle (EV) functions by the parent cell type, the authors organize evidence around anti- and pro-tumor responses. This mechanism-based review makes the exosome influence on cancer more accessible. They describe evidence, and the mechanistic underpinnings, for anti-tumor activities, including cytokine regulation, immune cell activation, macrophage reprogramming, checkpoint reversal, cytotoxicity, and inhibition of metastasis. They take a parallel approach to pro-tumor responses, addressing evidence of, and mechanisms behind, immunosuppression, excessive checkpoint signaling, and metastatic responses. Finally, the review turns its attention to translation, identifying that quality control, omics-based approaches, and artificial intelligence could help overcome technical challenges and accelerate the translational promise of EVs in cancer treatment.

Together, the findings presented or reviewed in these six articles collectively demonstrate how advances in immunology are uncovering key mechanisms that govern immune responses in infection, inflammatory diseases, and cancer. These studies identify potential mechanisms and therapeutic targets, including sepsis-associated pathways, natural immunomodulators, antiviral regulators, tumor microenvironment modulation, combinatorial chemoimmunotherapeutic strategies incorporating DC vaccines, and exosome-mediated signaling, which may support the future development of innovative immunomodulatory therapies.

